# Novel rapid infusion device for patients in emergency situations

**DOI:** 10.1186/1757-7241-19-35

**Published:** 2011-06-10

**Authors:** Dheeraj Kapoor, Manpreet Singh

**Affiliations:** 1 Department of Anaesthesiology and Intensive Care, Govt. Medical College and Hospital, Sector 32, Chandigarh-160030, India

**Keywords:** Rapid infusion system, fluid administration, trauma

## Abstract

Rapid fluid administration is often required for resuscitation when patients are admitted in emergency department with hypovolemic shock or excessive blood loss. Various methods have been described earlier to increase the fluid administration speed. Larger vein size, larger bore cannula, height of fluid, pressure over fluid bottle etc. are some of methods described in such situations.

We here describe a novel method to administer intravenous fluid rapidly and this method can be utilized in emergency and trauma settings.

## Background

In prehospital trauma and emergency settings, the immediate establishment of venous access and rapid fluid administration may be difficult in resuscitation of patients in hypovolemic shock due to massive blood loss. Rapid infusion systems (RIS) have been successfully used for delivering large amount of intravenous fluids at standard and rapid flow rates. Although RIS are undoubtedly the convenient and most effective way of delivering fluids in short span of time but it has its own limitations.

**Figure 1:**
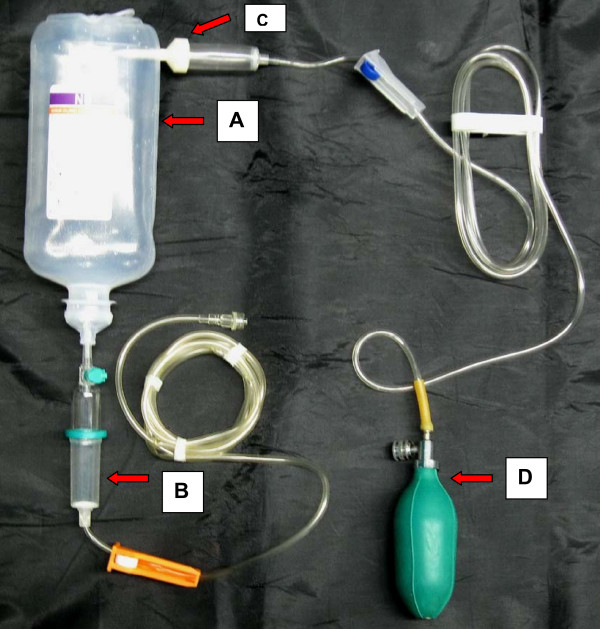
**Shows the assembly of RIS**. A: Fluid bottle B: Airtrap containing chamber C: Another drip set is put above fluid level D: Self inflating bag

We present a novel device which can be successfully used in prehospital trauma and emergency settings. The steps for designing this equipment are as follows (Figure 1):

1. A collapsible bag/Thin plastic intravenous fluid bottle is taken (A)

2. The sterile Intrafix^® ^safeset (M/S B. Braun Melsungen, Germany) is inserted in the intravenous (IV) fluid bottle and the other end is connected to the patient's intravenous cannula (B).

3. The spike end of intravenous set is inserted above fluid level in fluid bottle(C)

4. The sphygmomanometer inflation bulb is connected to latex end of intravenous tubing (that is inserted in fluid bottle) (D).

5. The roller clamps of both IV tubing's are rolled ON.

## Functional physics

By inflating the inflation bulb of the sphygmomanometer the air is pushed in the fluid bottle. This creates a vertical pressure head on the surface of the fluid column and along with gravitational force, it pushes fluid through the fluid bottle along the IV tubing, to the patient. The Intrafix^® ^safe set (M/S B. Braun Melsungen, Germany) IV infusion assembly is used for infusing fluids to the patient.

## Discussion

In the past, there were many methods used for rapid delivery of fluids. Amongst them various pressurization techniques like gravity-fed infusion, manual compression of fluid chamber, and compression of fluid chamber using flexible (Infusable Disposable Pressure Infuser, Vital Sign Inc., Tatowa, NJ, USA) and rigid pressure bags (Norfolk and Norwich medical equipment, Norwich, UK), were popularly used. The efficacies of these techniques to increase the flow rates were also evaluated. They found that manual push-pull technique was better than gravity fed infusion system [1,2].Pressure bags tend to increase the flow rates significantly and were found to be an effective method for rapid fluid infusions [3,4].Various other methods were also used like multiple fluid infusions, reducing the length of IV cannula [5], large bore catheters inserted in major veins [6,7] and manual injection of fluid [8], to obtain desired result.

Presently, rapid infusion systems (Haemonetics Corp., Braintree, MA) are commonly used to administer blood and fluids at desired and rapid rates. These rapid infusion devices are large, bulky, expansive, and costly to operate. All of these devices are operated by large, heavy, non-portable, roller pump mechanism and require fresh sets of fluid administration each time. These devices cannot be used with typical peripheral IV cannula but require large-bore central-line or venous cut-down catheters which can be inserted only by experts. Although rapid infusion devices are a proven life saver but this technology is not commonly accessible most of the hospitals in developing countries because of the aforementioned reasons. Further, the modern RIS are bulky and costly that makes their use difficult and cumbersome in emergency and disaster scenarios.

The rapid infusion device assembly we describe solves many problems present in prior art devices. It is small, portable, and if desired can be designed easily by any health care provider in any type of set-up. It is inexpensive, environment friendly and can be potentially available to patients even in small rural hospitals. This can be used with any IV tubing or other commonly available hospital equipment and can be used with central lines, venous cut-down catheters, or peripheral IV s that nurses or paramedics can insert. Therefore, it has a potential application for use in ambulances, in the fields, in emergency rooms, military applications and camp surgeries in disasters.

We are routinely using this device successfully in our institution in ambulances and ER settings for immediate volume resuscitation without any complication of air embolism or hemolysis. We used Intrafix^® ^safeset (M/S B. Braun Melsungen, Germany) IV infusion assembly for infusing fluids instead of conventional IV tubing assembly, as this IV infusion set has a unique airtight hydrophilic filter membrane (pore size 15 μm) with an air barrier integrated into the drip chamber which allows only fluid to pass and thereby prevents the inflow of air into the tubing. This acts as a safety feature against air passing through the IV tubing thus preventing chances of air embolism. It also negates the need of pushing the air bubble up and out of the bottle and priming of the drip chamber while switching of the bottle with air in the infusion system. Thus further reduces the time of administration as compared to the conventional IV tubing, thus ideal for rapid infusion of fluids in emergency situations.

We strongly recommend that rapid infusion of fluids using this assembly can be used as an effective alternative to traditional and standard methods especially in prehospital and peripheral setups for rapid volume resuscitation.

## Competing interests

The authors declare that they have no competing interests.

## Authors' contributions

DK designed this device along with 2^nd ^author MS.

MS wrote this manuscript with corrections by DK.Both authors read and approved the final manuscript.
